# Chromosome painting and phylogenetic analysis suggest that the genus *Lophostoma* (Chiroptera, Phyllostomidae) is paraphyletic

**DOI:** 10.1038/s41598-022-21391-1

**Published:** 2022-11-14

**Authors:** Natalia Karina Nascimento da Silva, Cleusa Yoshiko Nagamachi, Luis Reginaldo Ribeiro Rodrigues, Patricia Caroline Mary O’Brien, Fengtang Yang, Malcolm Andrew Ferguson-Smith, Julio Cesar Pieczarka

**Affiliations:** 1 Laboratório de Citogenética, Centro de Estudos Avançados da Biodiversidade, Instituto de Ciências Biológicas, Universidade Federal do Pará (UFPA), Belém, Pará Brazil; 2 Departamento de Morfofuncional, Universidade do Estado do Pará, Tucuruí, Pará Brazil; 3 Laboratório de Genética & Biodiversidade, Instituto de Ciências da Educação, Universidade Federal do Oeste do Pará, Santarém, Pará Brazil; 4 Department of Veterinary Medicine, Cambridge Resource Centre for Comparative Genomics, University of Cambridge, Cambridge, UK; 5 Cytogenetics Facility, Wellcome Trust Sanger Institute, Hinxton, UK; 6 School of Life Sciences and Medicine, Shandong University, Jinan, China

**Keywords:** Phylogenetics, Evolutionary genetics, Cytogenetics, Evolutionary biology

## Abstract

The subfamily Phyllostominae (Chiroptera, Phyllostomidae) comprises 10 genera of Microchiroptera bats from the Neotropics. The taxonomy of this group is controversial due to incongruities in the phylogenetic relationships evident from different datasets. The genus *Lophostoma* currently includes eight species whose phylogenetic relationships have not been resolved. Integrative analyzes including morphological, molecular and chromosomal data are powerful tools to investigate the phylogenetics of organisms, particularly if obtained by chromosomal painting. In the present work we performed comparative genomic mapping of three species of *Lophostoma* (*L. brasiliense* 2n = 30, *L. carrikeri* 2n = 26 and *L. schulzi* 2n = 26), by chromosome painting using whole chromosome probes from *Phyllostomus hastatus* and *Carollia brevicauda*; this included mapping interstitial telomeric sites. The karyotype of *L. schulzi* (LSC) is a new cytotype. The species *L. brasiliense* and *L. carrikeri* showed interstitial telomeric sequences that probably resulted from expansions of repetitive sequences near pericentromeric regions. The addition of chromosomal painting data from other species of Phyllostominae allowed phylogeny construction by maximum parsimony, and the determination that the genera of this subfamily are monophyletic, and that the genus *Lophostoma* is paraphyletic. Additionally, a review of the taxonomic status of LSC is suggested to determine if this species should be reclassified as part of the genus *Tonatia*.

## Introduction

The subfamily Phyllostominae (Chiroptera, Phyllostomidae) is composed of 10 genera with 25 species^1^ that are organized into three tribes: Phyllostomini (*Phyllostomus*, *Tonatia*, *Mimon*, *Gardnerycteris*, *Phylloderma*, *Lophostoma*), Macrophyllini (*Trachops*, *Macrophyllum*) and Vampyrini (*Chrotopterus*, *Vampyrum*)^2^. The phylogenetic relationships between the genera of this subfamily and even their positioning in relation to other subfamilies of Phyllostomidae are controversial, as different data sets show different phylogenetic patterns, which result in different taxonomic classifications [for review:^2–7^].

The genus *Lophostoma* d’Orbigny, 1836 includes eight species: *L. silvicola* d’Orbigny, 1836; *L. brasiliense* Peters, 1867; *L. carrikeri* Allen 1910; *L. evotis* Davis & Carter 1978; *L. occidentalis* Davis & Carter 1978; *L. schulzi* Genoways & Williams 1980; *L. yasuni* Fonseca & Pinto, 2004 and *L. kalkoae* Velazco & Gardner 2012^8^, which range from southern Mexico to central Paraguay^9,10^.

Morphological, molecular and chromosomal data, analyzed under an integrative taxonomy approach, constitute powerful tools for understanding the phylogenetic relationships between groups of organisms. Comparative analysis by chromosomal painting between species, associated with chromosome banding, can elucidate the types of intraspecific and interspecific rearrangements involved in the process of chromosomal differentiation that occurred throughout the evolution of taxa^11–13^. Chromosomal painting data have been successfully used in the reconstruction of karyotypic evolution among several groups of bats, including Phyllostomidae, which resulted in the correct identification of chromosomal homologies, the reconstruction of the phylogeny of the family and interpretation of the ancestral karyotype^14–22^.

Species of the genus *Lophostoma* show variable rates of karyotypic evolution where some species have highly conserved karyotypes such as *L. silvicola* (2n = 34, NF = 60; Gardner; Honeycutt et al.; Ribas et al.)^20,23,24^ and *L occidentalis* (2n = 34, NF = 62)^16,25^ On the other hand, the genus also has species with highly rearranged karyotypes: *L. brasiliense* (2n = 30, NF = 56)^23,26–29^, *L. carrikeri* (2n = 26, NF = 46)^23,30^ and *L. schulzi* (2n = 28, NF = 36)^24,30,31^. Analyzing the species *L. schulzi*, Baker et al.^29^ found a karyotype so derived that none of the chromosomal arms proposed as primitive for the family were identified, suggesting that non-Robertsonian rearrangement processes would be involved in the differentiation of these karyotypes. In addition, most of the karyotypes were presented only in Giemsa conventional staining, preventing comparisons that could provide information about the types of chromosomal rearrangements that distinguish them.

In the present work, the karyotypes of three species of the genus *Lophostoma* (*L. schulzi*, *L. brasiliense* and *L. carrikeri*) were analyzed by chromosome painting using whole chromosome probes from *Phyllostomus hastatus* and *Carollia brevicauda*^17^. Chromosomal painting data for *L. occidentalis*^16^, *L. silvicola*^20^, *Phyllostomus hastatus*^17^, *Gardnerycteris crenulatum*^16^, *Tonatia bakeri*^16^, *Tonatia maresi*^20^, and *Macrotus californicus*^15^, were added to the phylogenetic analysis, in order to observe the relationships between *Lophostoma* species and their position within the Phyllostomini tribe.

## Results

### Karyotypic description and FISH in *Lophostoma schulzi* (LSC)

**Figure 1:**
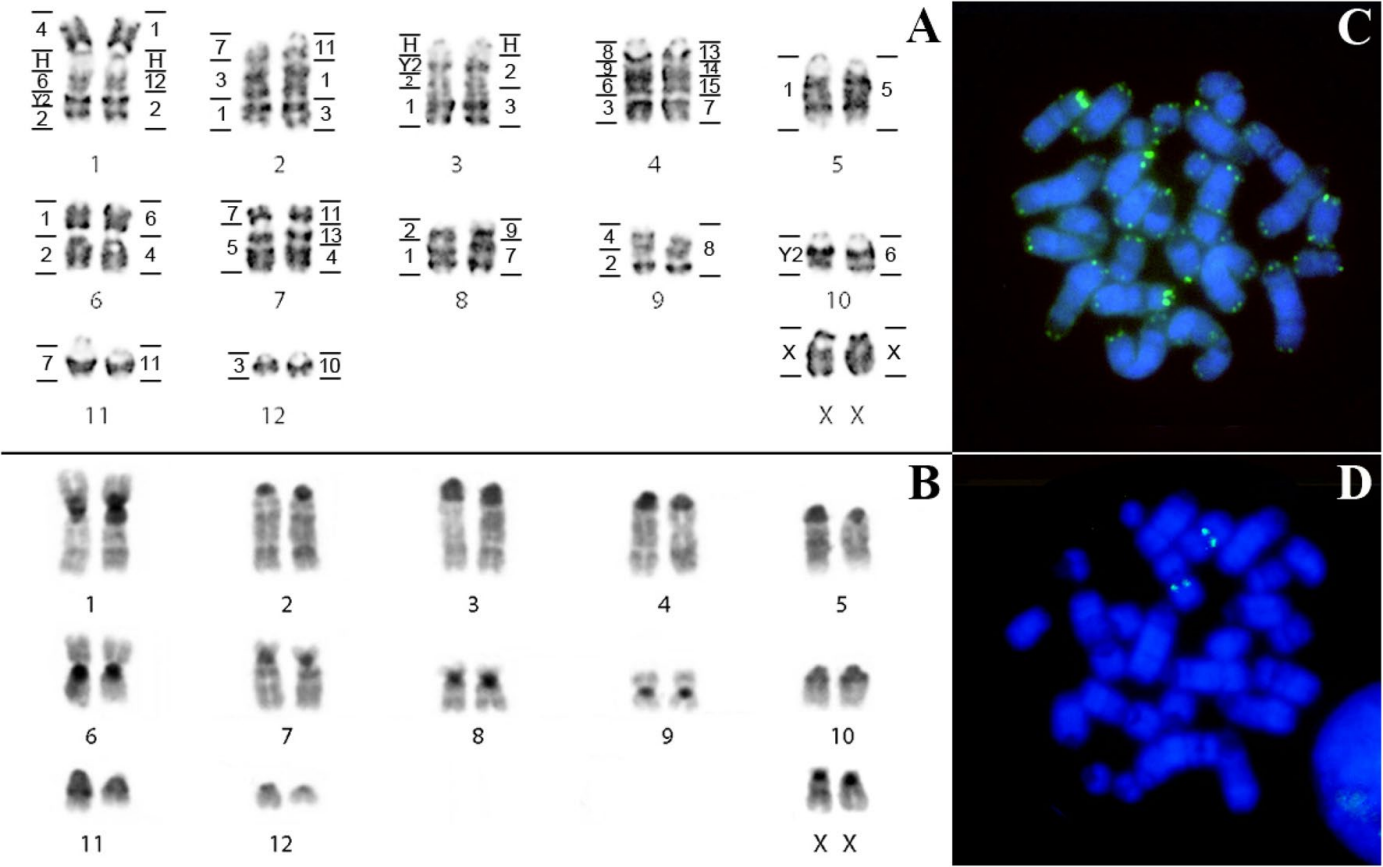
*Lophostoma schulzi* (LSC): (**A**) Karyotype with G-banding. The horizontal lines on the right of each pair of chromosomes delimit the hybridization with whole chromosome probes from *Phyllostomus hastatus* and the horizontal lines on the left, the probes from *Carollia brevicauda*. H identifies the amplified heterochromatic regions. (**B**) C-banding pattern showing constitutive heterochromatin regions. (**C**) FISH with telomeric probes. (**D**) FISH with 18S ribosomal DNA probe.

*Lophostoma schulzi* shows 2n = 26 NF = 34, comprising metacentric (pairs 6 and 9), submetacentric (pair 1), subtelocentric (7 and 8) and 7 acrocentric pairs (2, 3, 4, 5, 10, 11 and 12). The X chromosome presents acrocentric morphology (Fig. 1A). Constitutive heterochromatin (CH) occurs in the pericentromeric region of all chromosomes, and more extensive heterochromatic blocks were observed in pairs 1, 3, 6, 8, 10 and 11 (Fig. 1B). In situ hybridization with telomeric probe occurred only at the telomeres of all chromosomes (Fig. 1C). In situ hybridization with 18S ribosomal DNA probes confirmed the location of the NOR in pair 6 (Fig. 1D). Chromosomal painting using *Phyllostomus hastatus* (PHA) and *Carollia brevicauda* (CBR) probes delimited 24 and 39 homologous segments in the genome of *Lophostoma schulzi* respectively. The number of signals per chromosome pair ranged from one to five.

### Karyotypic description and FISH in *Lophostoma brasiliense* (LBR)

**Figure 2:**
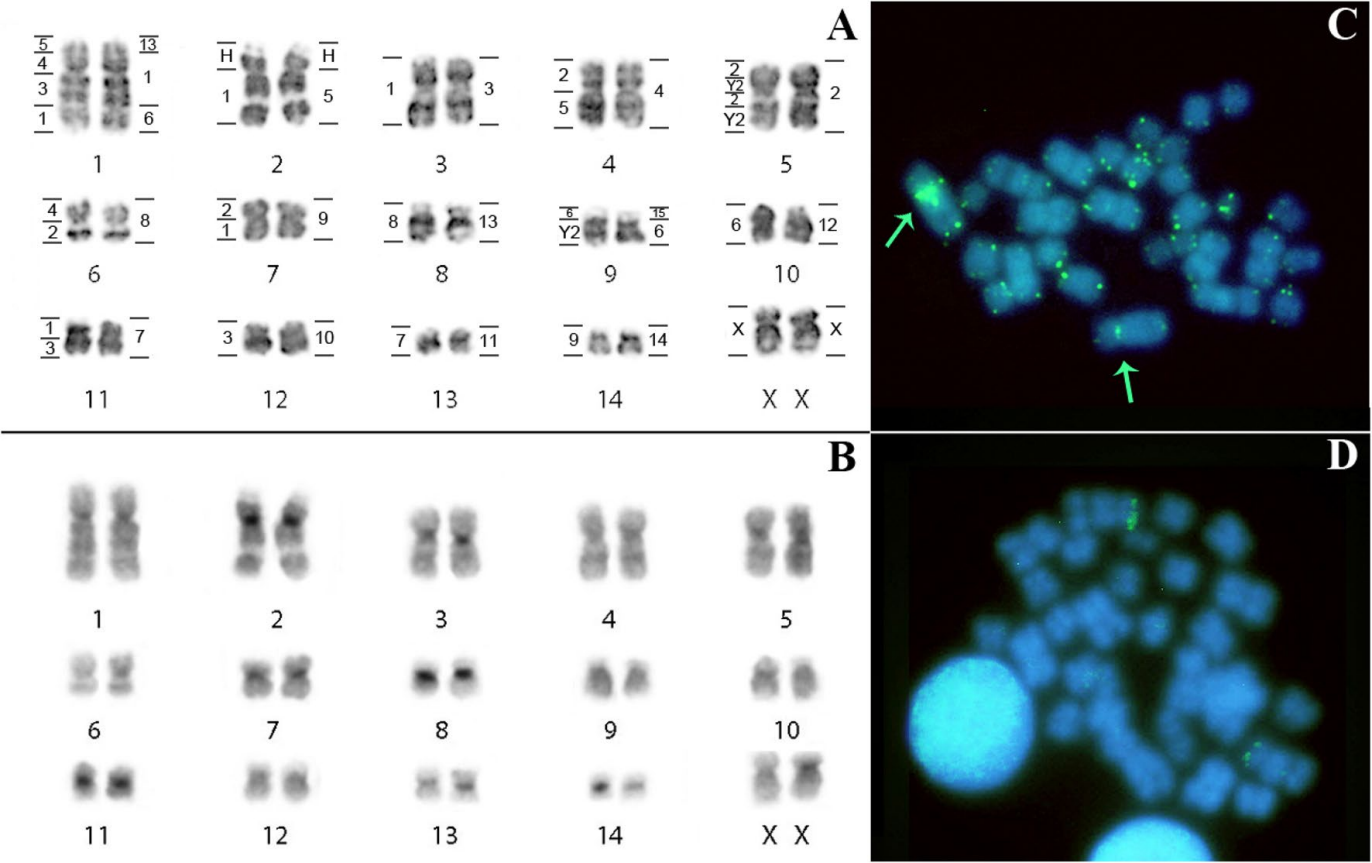
*Lophostoma brasiliense* (LBR): (**A**) Karyotype with G-banding. The horizontal lines to the right of each pair of chromosomes delimit the hybridization with whole chromosome probes from *Phyllostomus hastatus* and the horizontal lines on the left, the probes from *Carollia brevicauda*. H identifies the amplified heterochromatic regions. (**B**) C-banding pattern showing constitutive heterochromatin regions. (**C**) FISH with telomeric probes. Arrows: interstitial telomeric sequences. (**D**) FISH with 18S ribosomal DNA probe.

*Lophostoma brasiliense* shows 2n = 30 NF = 56, and the autosomal complement consists of 9 pairs of metacentric chromosomes (pairs 4, 5, 6, 7, 8, 11, 12, 13 and 14), three submetacentric pairs (pairs 1, 2, 3) and two subtelocentric pairs (pairs 9 and 10). The X chromosome is subtelocentric of medium size (Fig. 2A). The C-banding technique demonstrated that constitutive heterochromatin is located in the pericentromeric region of all autosomal and sex chromosomes (Fig. 2B). In situ hybridization with telomeric probe showed distal markings, common to the telomeres of all chromosomes, and the detection of an interstitial telomeric sequence (ITS) in pair 1 (Fig. 2C). FISH with 18S rDNA probes showed that the NOR is located in the distal region of the short arm of pair 2 (Fig. 2D). Chromosomal painting using PHA and CBR probes delimited 17 and 24 homologous segments in the genome of *Lophostoma brasiliense*, respectively. The number of signals per chromosome pair ranged from one to four.

### Karyotypic description and FISH in *Lophostoma carrikeri* (LCA)

**Figure 3:**
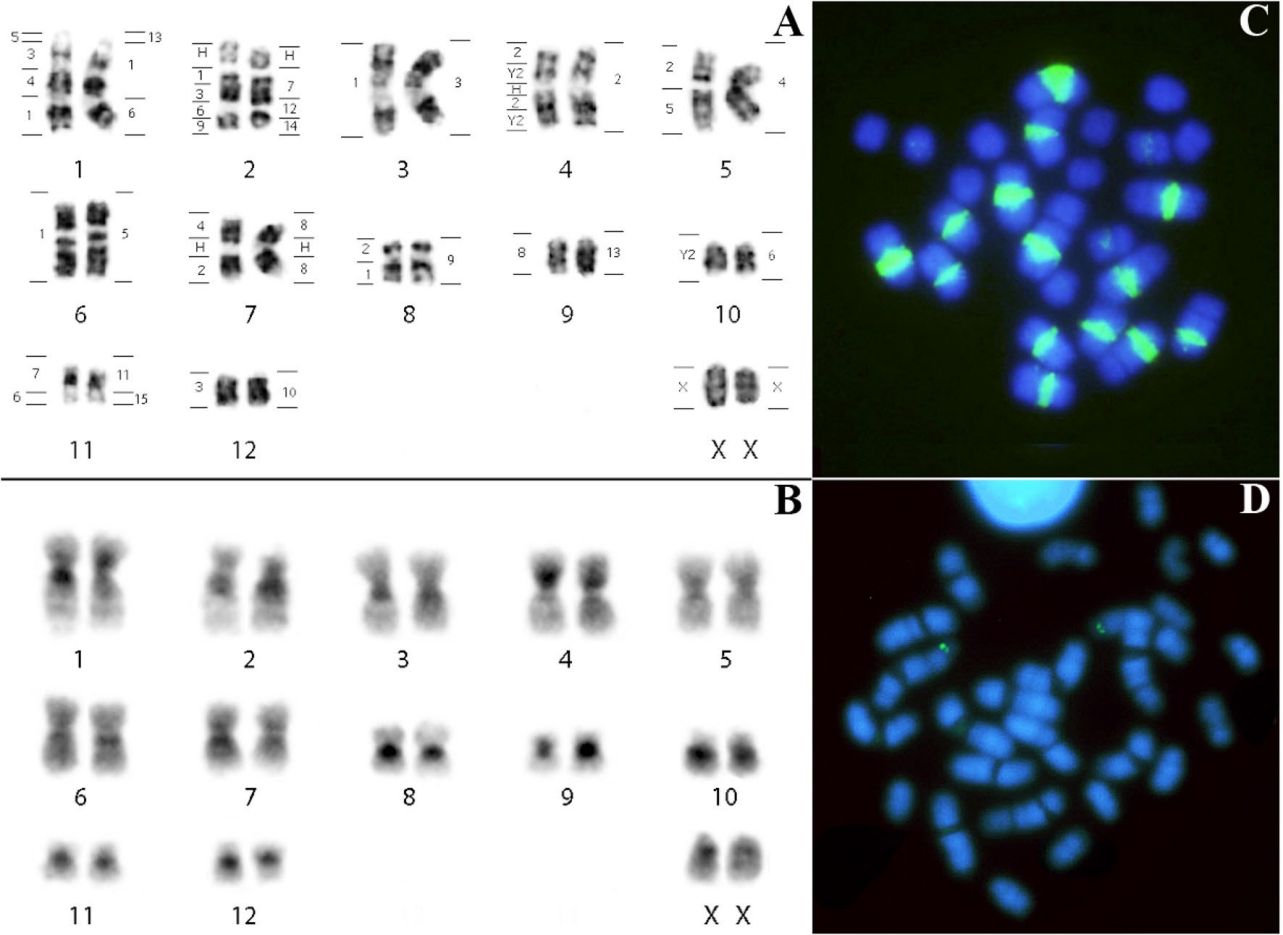
*Lophostoma carrikeri* (LCA): (**A**) Karyotype with G-banding. The horizontal lines to the right of each pair of chromosomes delimit the hybridization with whole chromosome probes from *Phyllostomus hastatus* and the horizontal lines on the left, the probes from *Carollia brevicauda*. H identifies the amplified heterochromatic regions. (**B**) C-banding pattern showing constitutive heterochromatin regions. (**C**) FISH with telomeric probes. (**D**) FISH with 18S ribosomal DNA probe.

*Lophostoma carrikeri* had a diploid number 2n = 26 NF = 48, comprising 10 metacentric pairs of chromosomes (pairs 3, 4, 5, 6, 7, 8, 9, 10, 11 and 12), and two large subtelocentric pairs (pairs 1 and 12). The X chromosome showed acrocentric morphology (Fig. 3A). Constitutive heterochromatin occurs in the pericentromeric region in all autosomes and in the X (Fig. 3B). In situ hybridization with telomeric probe occurred at telomeres and in the pericentromeric region in seven pairs of metacentric chromosomes exhibiting strong signals co-localized to heterochromatic blocks identified by the C-banding pattern (Fig. 3C). In situ hybridization with 18S ribosomal DNA probes identified the location of the NOR on the short arm of pair 2 (Fig. 3D). Chromosome painting using PHA and CBR probes delimited 19 and 26 homologous segments in the genome of *Lophostoma carrikeri*, respectively. The number of signals per chromosome pair ranged from one to four. No hybridization signal was found in the short arm of the subtelocentric pair 2, which was shown to be heterochromatic by C-banding.

**Figure 4:**
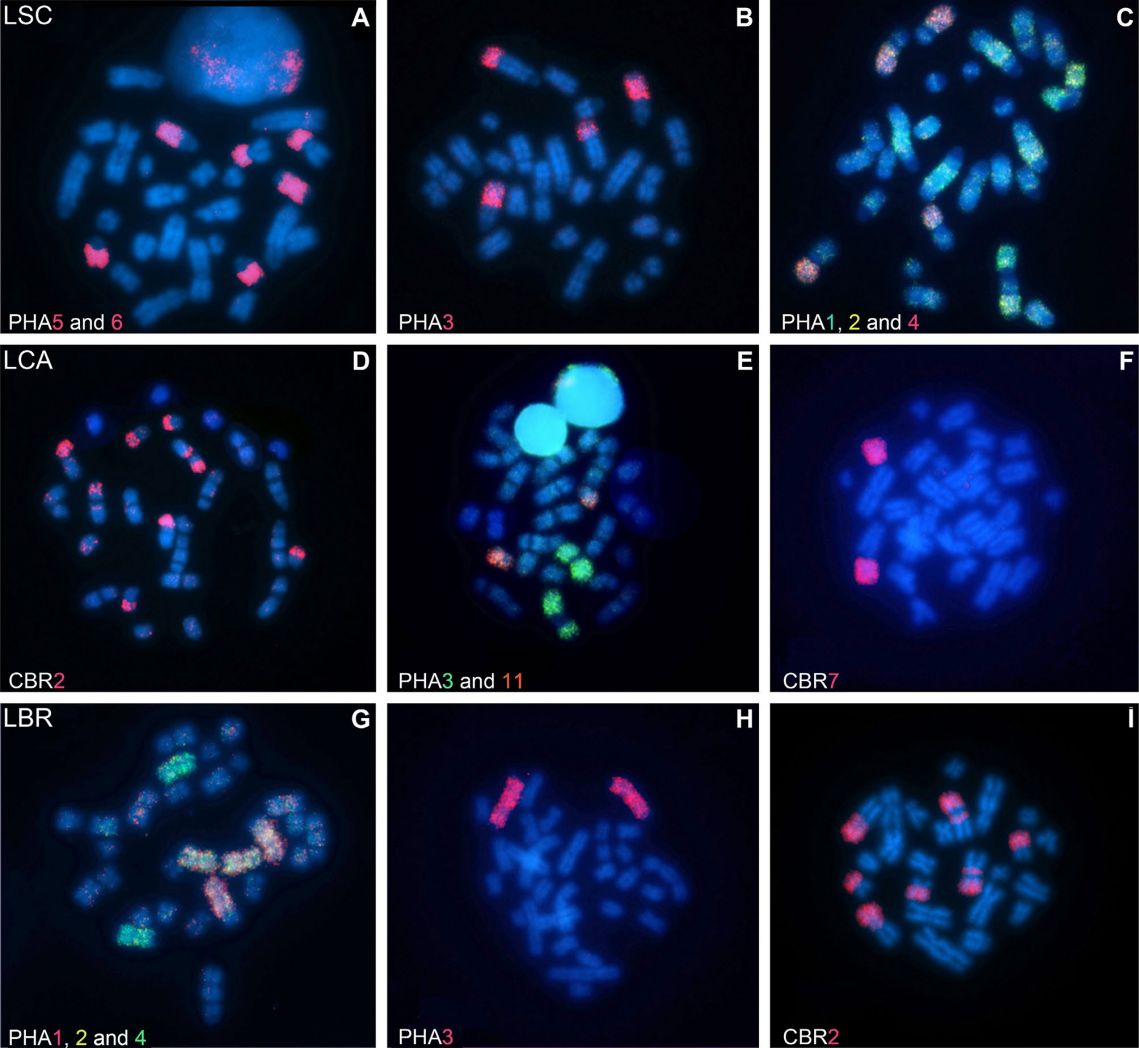
Representative images of fluorescence in situ hybridizations with whole chromosome probes from *Phyllostomus hastatus* (PHA) and *Carollia brevicauda* (CBR) in metaphases of species of *Lophostoma*. The probes used in LSC (**A–C**), LCA (**G–I**) and LBR (**D–F**) are indicated in white in the lower corner of each image.

Some examples of the results of hybridizations made in the metaphases of the three species studied are shown in Fig. 4.

### Phylogenetic analysis

**Figure 5:**
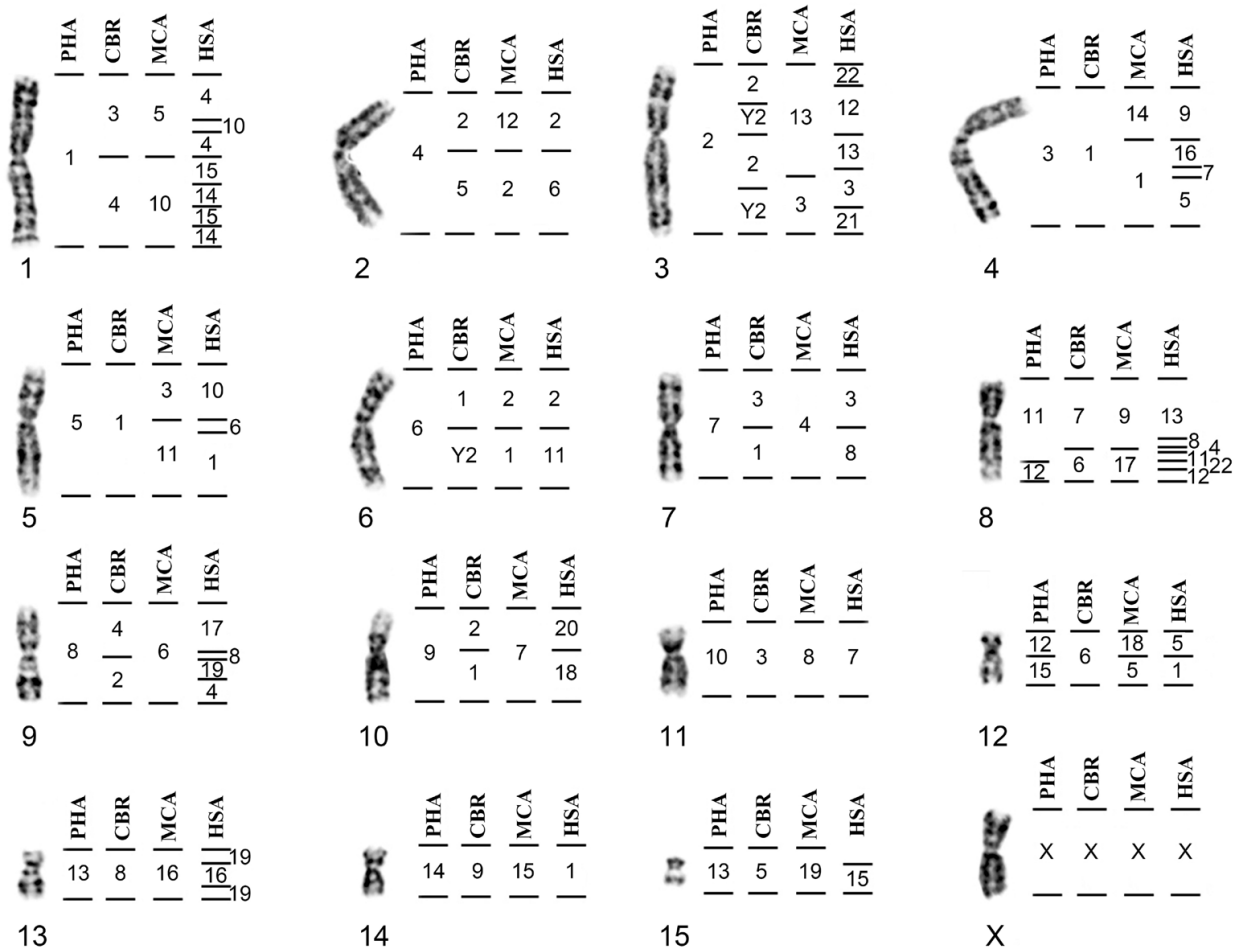
Homeology of the syntenic groups of PHA and CBR^21^ with HSA and MCA^15^ using GSO chromosomes as reference. This figure is complementary to Fig. 3 in Ref.^21^.

**Figure 6:**
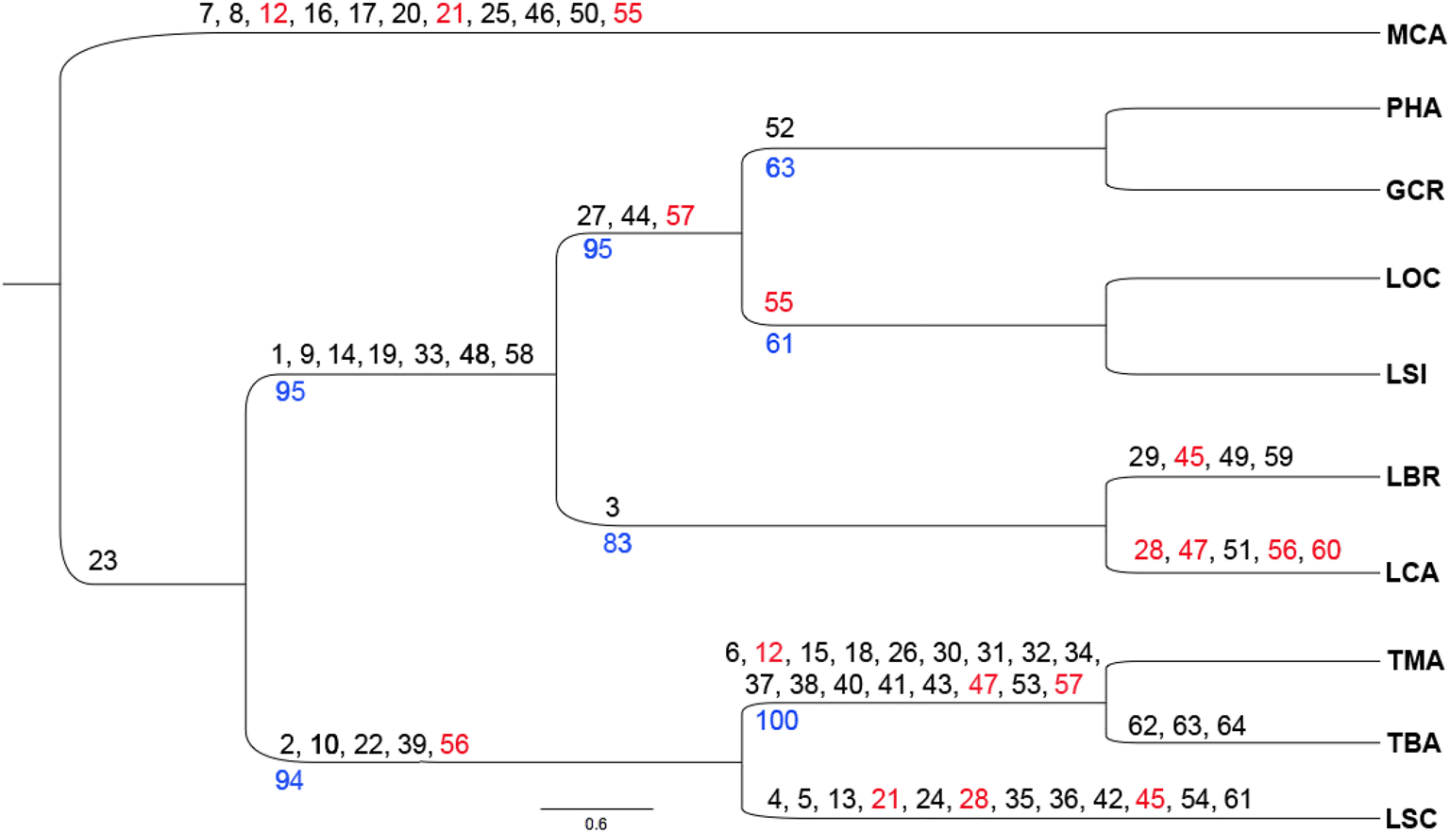
Phylogenetic tree obtained using chromosomal characters from maximum parsimony in the PAUP program for representatives of the genus *Lophostoma*. The blue numbers below the branches represent bootstrap values. The numbers in black refer to the apomorphies described in Supplementary Table 1. The numbers in red refer to the apomorphies that present homoplasy and are repeated in the branches in which they occur. *MCA Macrotus californicus*; *PHA Phyllostomus hastatus*, *GCR Gardnerycteris crenulatum*, *LOC Lophostoma occidentalis*, *LSI Lophostoma silvicola*, *LBR Lophostoma* brasiliense, *LCA Lophostoma carrikeri*, *TMA Tonatia maresi*, *TBA Tonatia bakeri*, *LSC Lophostoma schulzi*.

The chromosome paintings by Sotero-Caio et al.^15,16^ were made using whole chromosome probes from *Macrotus californicus*. Once we mapped the *Glossophaga soricina* genome with the PHA and CBR whole chromosome probes^21^ and Sotero-Caio et al.^16^ mapped the same species with human and MCA probes, it was possible to compare the distribution of the probes and determine their homeology (Fig. 5). For the purposes of our phylogenetic analysis, we converted the information from MCA to PHA (Supplementary Table 1) based on Fig. 5, in order to build a unified data matrix. A single tree was recovered by our maximum parsimony analysis (Fig. 6). The Phyllostomini genera formed a monophyletic group. The genus *Lophostoma*, however, is paraphyletic. One of the branches brings together the species of *Tonatia* and *L. schulzi*. The other branch aggregates the other species studied here, where one of the branches brings together *L. brasiliense* and *L. carrikeri*; the other branch is divided in two, being *L. occidentalis* and *L. silvicola* in one of the branches and *Phyllostomus hastatus* and *Gardnerycteris crenulatum* in the other branch. The consistency index (CI) was 0.88, the retention index (RI) = 0.8594 and the homoplasy index (HI) = 0.12. Bootstrap values ranged from 61 to 100.

## Discussion

### Chromosomal differences in *Lophostoma*

The data on the karyotype of the specimens of *Lophostoma brasiliense* (2n = 30 NF = 56) and *Lophostoma carrikeri* (2n = 26 NF = 46) analyzed here are in agreement with the studies previously carried out^23^, in specimens collected in Peru and for specimens collected in Suriname^24,29,32^. We described a new cytotype for *Lophostoma schulzi* 2n = 26 (6 sm/4 st + 16 a) for individuals collected in Juruti, State of Pará, Brazil (present study) that differs from the *L. schulzi* cytotype 2n = 28 (4 sm/6 st + 18 a), for individuals collected in Suriname^24,30^. Comparative analysis between *L. schulzi* karyotypes from Suriname and Brazil showed that the difference is due to a centric fusion/fission rearrangement. Chromosomal variations for populations of this species had not yet been reported, although they have already been recorded for other phyllostomid^19,29,33–36^.

Most Phyllostomidae species have a bi-armed X chromosome, and this condition is considered basal for the family^28,37,38^. The acrocentric form of X has been reported only for some genera, such as *Micronycteris* and *Mesophylla*^26–28^, a form admitted as a homoplasic character in these species because they are not closely related. In *Lophostoma*, the acrocentric X chromosome is found only in *L. carrikeri* and *L. schulzi* [Refs.^16,20,24,25^, present work], similarly suggesting a homoplasic character, since these species are phylogenetically far apart (Fig. 6).

### Distribution of heterochromatin and telomeric sites in *Lophostoma*

A pericentromeric pattern of constitutive heterochromatin distribution was observed in *L. brasiliense* (Fig. 2B), which is commonly found in phyllostomid bats^38–41^. Unusual distribution was observed in the karyotype of *L. schulzi* (Fig. 1B) and *L. carrikeri* (Fig. 3B), which have extensions of heterochromatin beyond the pericentromeric region.

The chromosomal location of telomeric sequences by FISH has been determined in many groups of animals^42–45^. In addition to distal markings, interstitial telomeric sites (ITS) were also reported^46–48^. Telomeric hybridization was observed here at the ends of all chromosomes of species of the genus *Lophostoma*. Additionally, ITS were observed in *L. brasiliense* (Fig. 2C) and *L. carrikeri* (Fig. 3C), where we identified strong signals co-located with heterochromatic blocks marked by the C-positive band pattern, which suggests that these sequences are present as satellite DNA components in the centromeric region and not as a result of fusion processes. However, the presence/absence of large C-positive band blocks is not directly related to the presence/absence of telomeric sequences. We found extensive blocks with positive C-banding in the karyotype of *L. schulzi*, but no ITS was observed in the individuals analyzed in this study, exemplifying the heterogeneity of the repetitive DNA that makes up the heterochromatic regions in the mammalian genome^46^.

### 18S ribosomal DNA location

When analyzing the location of the 18S ribosomal DNA in Phyllostomidae species mapped using chromosome painting and 18S rDNA probes^16,20–22^, it is very common to find its mapping in different places, an observation already found in literature^16^. Regarding the genus *Lophostoma*, five species were studied using these probes. In four, LCA, LBR, LSC (current study) and *L. silvicola* (LSI)^20^ the 18S ribosomal DNA mapped into a block of heterochromatin. The only exception is *L. occidentalis* (LOC)^16^, where the 18S rDNA maps to chromosomes corresponding to pairs 5 and 19 of *Macrotus californicus* (PH1 and PHA13, respectively, see Fig. 5). This seems to suggest that the 18S rDNA was located in a heterochromatic block in the *Lophostoma* ancestral karyotype. However, a DNA sequence study is necessary to verify whether all heterochromatic blocks harboring 18S DNA in *Lophostoma* are homologous.

### Comparative chromosomal mapping in species of the genus *Lophostoma*

Through chromosome painting, using PHA and CBR whole chromosome probes, it was possible to establish the homeologies between the karyotypes of the species analyzed here. Additionally, we added the data previously obtained with these same probes in *L. silvicola*^20^ and the analysis with MCA probes in *L. occidentalis*^16^, where we converted the MCA data to PHA, as illustrated in Fig. 5. *Lophostoma* karyotypes were highly variable, from highly conserved forms (LOC) to karyotypes with many rearrangements (LSC).

Considering the PHA karyotype as a reference, as it is close to the ancestral karyotype proposed for Phyllostomidae^18,22^, we show that the LOC and LSI karyotypes are very similar to the PHA karyotype, differing only by the fission of PHA13. In addition to presenting autapomorphies, the LBR and LCA karyotypes share the syntenic arrangement PHA13/1/6 (pair 1 in both species). These species and also LOC and LSI share with PHA the pairs PHA1, 2, 3, 4, 7, 12 and a metacentric X. Finally, the LSC karyotype is the most differentiated in relation to other species, as most chromosomes are acrocentric and show a greater number of rearrangements in relation to the ancestral and PHA karyotypes. These data confirm the observation by Baker et al.^29^, that LSC has a derived karyotype in which none of the chromosomal arms proposed as primitive for the family were identified by G-banding.

### *Tonatia bakeri* × *Tonatia maresi*

Ribas et al.^20^ mapped the *Tonatia saurophila* karyotype collected in the Brazilian Amazon with PHA and CBR whole chromosome probes. Sotero-Caio et al.^16^ mapped the same species, collected in Central America, with MCA whole chromosome probes. Recently the specimens of *T. saurophila* were reclassified^49^, considering only specimens from Jamaica as belonging to this species, while specimens located in Central America and northeast of South America were considered to belong to the species *Tonatia bakeri* (TBA), while specimens from the Amazon would belong to the species *Tonatia maresi* (TMA), with the Andes being the barrier that separates these new species. The presence of karyotypic differences could be an additional diagnostic element to define these species. Thus, we converted the probe mapping data from MCA to PHA in TBA by Sotero-Caio et al.^16^ using Fig. 5 and compared with the results we described earlier for TMA^20^. Sotero-Caio et al.^16^ had already noticed the difference in morphology of the TBA4 pair (equivalent to TMA6) between the karyotypes, suggesting a pericentric inversion. After comparing the mapping results of the two species, we found three chromosomal rearrangements between these karyotypes: (1) the difference between TBA4 and TMA6 is actually due to an insertion of a segment of the PHA10 chromosome (= MCA3) into the short arm of TBA4; (2) Insertion of a segment of the PHA7 chromosome (= MCA4) into the long arm of TBA3 (= TMA5); (3) Inversion in the long arm of TBA2 (= TMA4), where the syntenic group corresponding to PHA1 (= MCA5, MCA10) are joined in TMA and separated (MCA5 and MCA10) in TBA. Therefore, these differences reinforce the specific status of the Central American (TBA) and South American (TMA) taxa and were included in the data matrix (Supplementary Table 1) for the construction of the phylogeny (Fig. 6).

### Phylogenetic relationships

The genus *Lophostoma* is paraphyletic, as it includes PHA and GCR in the same branch as LOC and LSI, while the other *Lophostoma* representatives are located in more basal branches (Fig. 6). It is possible that this result is a consequence of the chromosomal conservatism of PHA, GCR, LOC and LSI, since the karyotypes of these two species of *Lophostoma* differ from PHA and GCR only by the fission of the PHA13 chromosome. Therefore, there would not be a phylogenetic signal significant enough to separate these genera, as already observed^20^. Regarding the internal branches, the association of PHA with GCR occurs because their karyotypes does not show any difference with the techniques used here. This close relationship had already been observed in other phylogenetic studies using molecular data^50–52^. Likewise, the sister species relationship between LOC and LSI is evidenced by the shared fission of PHA13. These two species are close, as LOC was considered a subspecies of LSI until it received its specific recognition^53^, again explaining the absence of rearrangements between their karyotypes. The branch joining LBR and LCA has a strong phylogenetic signal, composed of the PHA13/1/6 syntenic association exclusive to these species, although the karyotypes are not identical as in the case of LOC x LSI, due to LBR and LCA autapomorphies. The association of LBR with LCA has also been observed in molecular phylogeny studies^52,53^. Finally, the association between *Tonatia maresi*, *Tonatia bakeri* and *L. schulzi* results from the shared synapomorphies between these taxa, including PHA16q/3, PHA2p/12p, PHA4p/13p, and PHA9 fission. *Lophostoma* species were once considered part of the genus *Tonatia*, being later separated into the current genus^54^. This separation results from molecular studies that place the two taxa in different branches^50,51,54^. However, the presence of four synapomorphic associations uniting LSC, TBA and TMA reinforce the phylogenetic proximity of these species. Consequently, a review of the taxonomic status of LSC would be important considering the possibility of this species being reclassified as part of the genus *Tonatia*.

The results of the present analysis are broadly in line with other studies on the evolution of *Lophostoma*, with the exception of its paraphyly and the proximity of LSC to the genus *Tonatia*. Studies including the other tribes of Phyllostominae can be expected to shed light on the phylogenetic relationships found here.

## Materials and methods

### Ethics declarations

All experimental protocols were approved by the Ethics Committee from Para Federal University (Comitê de Ética Animal da Universidade Federal do Pará) under Permit 68/2015. All methods were carried out in accordance with relevant guidelines and regulations. All methods are reported in accordance with ARRIVE guidelines (https://arriveguidelines.org). The Cytogenetics Laboratory from Centro de Estudos Avançados da Biodiversidade (UFPA) has permit number 19/2003 from the Ministry of Environment for sample transport and permit 52/2003 for using the samples for research. Sample collections were authorized by Instituto Chico Mendes de Conservação da Biodiversidade (ICMBio) and Secretaria de Estado de Meio Ambiente do Pará (SEMA-PA) under permit 020/2005 (Registration: 207419).

### Analyzed specimens

Cytogenetic analyzes were performed on samples collected in the Brazilian Amazon region (Table 1). Bats were captured from natural populations with the aid of mist nets. Chromosomal preparations and tissue biopsies were sent to the Cytogenetics Laboratory of the Federal University of Pará, in Belém. The specimens were fixed in 10% formaldehyde, preserved in 70% ethanol and deposited in the mammal collection of the Museu Paraense Emilio Goeldi.

**Table 1:** Sample of the present study.

Species	Sample	Locality
*Lophostoma brasiliense*	2M, 2F	02° 23ʹ 12.1ʹʹ S; 57° 38ʹ 22.0ʹʹ W
*Lophostoma carrikeri*	1F	02° 28ʹ 6.3ʹʹ S; 55° 59ʹ 37.2ʹʹ W
*Lophostoma schulzi*	3F	02° 28ʹ 6.3ʹʹ S; 55° 59ʹ 37.2ʹʹ W

### Chromosomal preparations and banding

Chromosomal preparations were obtained by the direct bone marrow extraction method^55^. G-banding patterns were obtained using trypsin solution^56^, with subsequent incubation in saline solution (0.5 × SSC) at 60 °C and staining with Wright’s solution^57^. C-banding was performed according to literature^58^. Karyotypes were arranged in decreasing order of chromosome size.

### Fluorescence in situ hybridization (FISH)

FISH using telomeric probe labeled with digoxigenin (All Human Telomere—Oncor), and detected with FITC-Cy3 labelled antibody, was performed according to the protocol provided by the manufacturer. The 18S rDNA probes were amplified by PCR using the probes NXS1 and NS8, as described in literature^59^. The probes were labeled with dUTP-biotin labelled antibody by nick translation and later detected with avidin-Cy3 or FITC labelled antibodies.

Whole chromosome probes from *Carollia brevicauda* and *Phyllostomus hastatus*^17^ were amplified and labeled by DOP-PCR^60,61^ and hybridized following previously described^17,61^. Briefly, slides were incubated with pepsin enzyme solution, washed in 2× SSC solution and serially dehydrated with ethanol (70%, 90% and 100%). The slides were then aged in an oven at 65 °C for two hours, denatured in formamide 70%/2× SSC for 50 s and mounted with hybridization solution (14 µl of solution containing: 50% formamide, 2 × SSC, 10% dextran sulfate and 1–3 µl of PCR product) for three days. After hybridization and stringency washing, biotin-labeled probes were detected with avidin-Cy3 or avidin-FITC (1 µg/ml; Amersham). The slides were mounted with Vectashield antifading solution (Vector Lab) and DAPI (4ʹ,6-diamidino-2-phenylindole) and the images were captured with the aid of the Zeiss Axiocam CCD camera controlled by the Axiovision 3.0 software, coupled to a Zeiss Axioplan 2 microscope. Chromosomes were identified according to their morphology and banding patterns interpreted from DAPI (4ʹ,6-diamidino-2-phenylindole) staining images edited in grayscale format.

### Phylogenetic analysis

We used syntenic segments and shared chromosomal associations to establish a matrix of characters (Supplementary Table 1), which were coded based on presence or absence, to be used in the maximum parsimony analysis in PAUP Version 4a, build 169^62^. All characters were weighted with the same weight, based on the equal probability of occurrence of chromosomal rearrangements. We searched the most parsimonious phylogenetic tree, which was obtained using the exhaustive search. The robustness of each node was evaluated by bootstrap estimation of 1000 repetitions. *Macrotus californicus*^15^ was used as outgroup and as ingroup the three species studied here and *L. occidentalis*^16^, *L. silvicola*^20^, *Phyllostomus hastatus*^17^, *Gardnerycteris crenulatum*^16^, *Tonatia maresi*^20^, and *Tonatia bakeri*^16^.

## Supplementary Information

Supplementary Table 1.

## Data Availability

All relevant data are within the paper and in the Supplementary Table 1. Data can be requested from the corresponding author.

## Abbreviations

2n: Diploid number
a: Acrocentric chromosome
BAC: Bacterial artificial chromosome
CBR: *Carollia brevicauda*
CH: Constitutive heterochromatin
Cy3: Cyanine 3-methine
DAPI: 4',6-Diamidino-2-phenylindole
DOP-PCR: Degenerate oligonucleotide-primed polymerase chain reaction
dUTP: 2ʹ-Deoxyuridine 5ʹ-triphosphate
FISH: Fluorescence in situ hybridization
FITC: Fluorescein isothiocyanate
FN: Fundamental number
GCR: *Gardnerycteris crenulatum*
GSO: *Glossophaga soricina*
ITS: Interstitial telomeric sequences
LBR: *Lophostoma brasiliense*
LCA: *Lophostoma carrikeri*
LOC: *Lophostoma occidentalis*
LSC: *Lophostoma schulzi*
LSI: *Lophostoma silvicola*
MCA: *Macrotus californicus*
sm: Submetacentric chromosome
st: Subtelocentric chromosome
NOR: Nucleolar Organization Region
PAUP: Phylogenetic analysis using parsimony
PHA: *Phyllostomus hastatus*
rDNA: Ribosomal DNA
SSC: Saline sodium citrate
TBA: *Tonatia bakeri*
TMA: *Tonatia maresi*
