# Supplementary material for: Chromosome painting and phylogenetic analysis suggest that the genus Lophostoma (Chiroptera, Phyllostomidae) is paraphyletic
Source: Sci Rep. 2022 Nov 14;12:19514. doi: 10.1038/s41598-022-21391-1 (PMC9663435; doi:10.1038/s41598-022-21391-1)
Supplement: Supplementary file 1 — Supplementary Table 1. [file 41598_2022_21391_MOESM1_ESM.docx]

Supplementary Table 1. Basic data matrix for presence/absence of characters corresponding to the presence of synteny (1) and absence (0) used in the analysis of maximum parsimony. Number of characters: 64. Number of informative characters: 37. Assoc=associated; free: short and long arms as separated chromosomes; p=short arm; q=long arm; Inv=inversion. MCA= *Macrotus californicus*; PHA= *P. hastatus*, TSA = *Tonatia saurophila*, GCR= *Gardnerycteris* *crenulatum,* LOC= *Lophostoma occidentalis*, LSI= *L. silvicola*, LSC= *L. schulzi*, LBR= *L. brasiliense*, LCA= *L. carrikeri.*

|  | Characters | MCA | PHA | TMA | TBA | GCR | LOC | LSI | LSC | LBR | LCA |
| --- | --- | --- | --- | --- | --- | --- | --- | --- | --- | --- | --- |
| 1 | 1 as in PHA | 0 | 1 | 0 | 0 | 1 | 1 | 1 | 0 | 1 | 1 |
| 2 | Assoc. 1q/3 | 0 | 0 | 1 | 1 | 0 | 0 | 0 | 1 | 0 | 0 |
| 3 | Assoc. 13/1/6 | 0 | 0 | 0 | 0 | 0 | 0 | 0 | 0 | 1 | 1 |
| 4 | Assoc. 11/1q/3 | 0 | 0 | 0 | 0 | 0 | 0 | 0 | 1 | 0 | 0 |
| 5 | Assoc. 1/12/2 | 0 | 0 | 0 | 0 | 0 | 0 | 0 | 1 | 0 | 0 |
| 6 | Assoc. 1p/13/10/1p | 0 | 0 | 1 | 1 | 0 | 0 | 0 | 0 | 0 | 0 |
| 7 | Assoc. 1q/15 | 1 | 0 | 0 | 0 | 0 | 0 | 0 | 0 | 0 | 0 |
| 8 | 1q free | 1 | 0 | 0 | 0 | 0 | 0 | 0 | 0 | 0 | 0 |
| 9 | 2 as in PHA | 0 | 1 | 0 | 0 | 1 | 1 | 1 | 0 | 1 | 1 |
| 10 | Assoc. 2p/12p | 0 | 0 | 1 | 1 | 0 | 0 | 0 | 1 | 0 | 0 |
| 11 | 2q free | 1 | 0 | 0 | 0 | 0 | 0 | 0 | 0 | 0 | 0 |
| 12 | Assoc. 2/5 | 1 | 0 | 1 | 1 | 0 | 0 | 0 | 0 | 0 | 0 |
| 13 | Assoc. 2q/3p | 0 | 0 | 0 | 0 | 0 | 0 | 0 | 1 | 0 | 0 |
| 14 | 3 as in PHA | 0 | 1 | 0 | 0 | 1 | 1 | 1 | 0 | 1 | 1 |
| 15 | Assoc. 9q/3p/7p | 0 | 0 | 1 | 1 | 0 | 0 | 0 | 0 | 0 | 0 |
| 16 | 3q free | 1 | 0 | 0 | 0 | 0 | 0 | 0 | 0 | 0 | 0 |
| 17 | Assoc. 3/6 | 1 | 0 | 0 | 0 | 0 | 0 | 0 | 0 | 0 | 0 |
| 18 | Assoc. 1/3q/4q | 0 | 0 | 1 | 1 | 0 | 0 | 0 | 0 | 0 | 0 |
| 19 | 4 as in PHA | 0 | 1 | 0 | 0 | 1 | 1 | 1 | 0 | 1 | 1 |
| 20 | 4q free | 1 | 0 | 0 | 0 | 0 | 0 | 0 | 0 | 0 | 0 |
| 21 | Assoc. 4p/6p | 1 | 0 | 0 | 0 | 0 | 0 | 0 | 1 | 0 | 0 |
| 22 | Assoc. 4p/13p | 0 | 0 | 1 | 1 | 0 | 0 | 0 | 1 | 0 | 0 |
| 23 | 5 as in PHA | 0 | 1 | 1 | 1 | 1 | 1 | 1 | 1 | 1 | 1 |
| 24 | 5 acro | 0 | 0 | 0 | 0 | 0 | 0 | 0 | 1 | 0 | 0 |
| 25 | 5q free | 1 | 0 | 0 | 0 | 0 | 0 | 0 | 0 | 0 | 0 |
| 26 | Assoc. 5/6/14 | 0 | 0 | 1 | 1 | 0 | 0 | 0 | 0 | 0 | 0 |
| 27 | 6 as in PHA | 0 | 1 | 0 | 0 | 1 | 1 | 1 | 0 | 0 | 0 |
| 28 | 6q free | 0 | 0 | 0 | 0 | 0 | 0 | 0 | 1 | 0 | 1 |
| 29 | Assoc. 6q/15 | 0 | 0 | 0 | 0 | 0 | 0 | 0 | 0 | 1 | 0 |
| 30 | Assoc. 6q/7q/8p | 0 | 0 | 1 | 1 | 0 | 0 | 0 | 0 | 0 | 0 |
| 31 | Assoc. 6q/11 | 0 | 0 | 1 | 1 | 0 | 0 | 0 | 0 | 0 | 0 |
| 32 | Assoc. 6p/14 | 0 | 0 | 1 | 1 | 0 | 0 | 0 | 0 | 0 | 0 |
| 33 | 7 as in PHA | 1 | 1 | 0 | 0 | 1 | 1 | 1 | 0 | 1 | 1 |
| 34 | Assoc. 7q/5 | 0 | 0 | 1 | 1 | 0 | 0 | 0 | 0 | 0 | 0 |
| 35 | Assoc. 7q/9p | 0 | 0 | 0 | 0 | 0 | 0 | 0 | 1 | 0 | 0 |
| 36 | Assoc. 7/15/14/13 | 0 | 0 | 0 | 0 | 0 | 0 | 0 | 1 | 0 | 0 |
| 37 | 8 as in PHA | 1 | 1 | 0 | 0 | 1 | 1 | 1 | 1 | 1 | 1 |
| 38 | Assoc. 8q/9p | 0 | 0 | 1 | 1 | 0 | 0 | 0 | 0 | 0 | 0 |
| 39 | 9 as in PHA | 1 | 1 | 0 | 0 | 1 | 1 | 1 | 0 | 1 | 1 |
| 40 | Assoc. 9p/13q | 0 | 0 | 1 | 1 | 0 | 0 | 0 | 0 | 0 | 0 |
| 41 | 10 as in PHA | 1 | 1 | 0 | 0 | 1 | 1 | 1 | 1 | 1 | 1 |
| 42 | 10inv | 0 | 0 | 0 | 0 | 0 | 0 | 0 | 1 | 0 | 0 |
| 43 | Assoc. 10q/12q | 0 | 0 | 1 | 1 | 0 | 0 | 0 | 0 | 0 | 0 |
| 44 | 11 as in PHA (subm) | 0 | 1 | 0 | 0 | 1 | 1 | 1 | 0 | 0  0 | 0 |
| 45 | 11inv acro | 0 | 0 | 0 | 0 | 0 | 0 | 0 | 1 | 1 | 0 |
| 46 | 11inv meta | 1 | 0 | 0 | 0 | 0 | 0 | 0 | 0 | 0 | 0 |
| 47 | Assoc. 11/15 | 0 | 0 | 1 | 1 | 0 | 0 | 0 | 0 | 0 | 1 |
| 48 | 12 as in PHA | 0 | 1 | 0 | 0 | 1 | 1 | 1 | 0 | 1 | 1 |
| 49 | 12inv | 0 | 0 | 0 | 0 | 0 | 0 | 0 | 0 | 1 | 0 |
| 50 | 12 diss. free | 1 | 0 | 0 | 0 | 0 | 0 | 0 | 0 | 0 | 0 |
| 51 | Assoc. 12q/14 | 0 | 0 | 0 | 0 | 0 | 0 | 0 | 0 | 0 | 1 |
| 52 | 13 as in PHA | 0 | 1 | 0 | 0 | 1 | 0 | 0 | 0 | 0 | 0 |
| 53 | Assoc. 13/15 | 0 | 0 | 1 | 1 | 0 | 0 | 0 | 0 | 0 | 0 |
| 54 | Assoc. 13/14 | 0 | 0 | 0 | 0 | 0 | 0 | 0 | 1 | 0 | 0 |
| 55 | 13 diss. free | 1 | 0 | 0 | 0 | 0 | 1 | 1 | 0 | 0 | 0 |
| 56 | 14 as in PHA | 1 | 1 | 0 | 0 | 1 | 1 | 1 | 0 | 1 | 0 |
| 57 | 15 as in PHA | 0 | 1 | 1 | 1 | 1 | 1 | 1 | 0 | 0 | 0 |
| 58 | X as PHA (meta) | 1 | 1 | 1 | 1 | 1 | 1 | 1 | 0 | 0 | 0 |
| 59 | X submeta | 0 | 0 | 0 | 0 | 0 | 0 | 0 | 0 | 1 | 0 |
| 60 | X acro | 0 | 0 | 0 | 0 | 0 | 0 | 0 | 0 | 0 | 1 |
| 61 | X subtelo | 0 | 0 | 0 | 0 | 0 | 0 | 0 | 1 | 0 | 0 |
| 62 | Inv in TBA2 | 0 | 0 | 0 | 1 | 0 | 0 | 0 | 0 | 0 | 0 |
| 63 | Ins in TBA4 | 0 | 0 | 0 | 1 | 0 | 0 | 0 | 0 | 0 | 0 |
| 64 | Ins in TBA3 | 0 | 0 | 0 | 1 | 0 | 0 | 0 | 0 | 0 | 0 |
